# Supplementary figures and images for: A potential photo-protective, antioxidant function for DMSO in marine phytoplankton
Source: PLoS One. 2025 Feb 6;20(2):e0317951. doi: 10.1371/journal.pone.0317951 (PMC11801556; doi:10.1371/journal.pone.0317951)

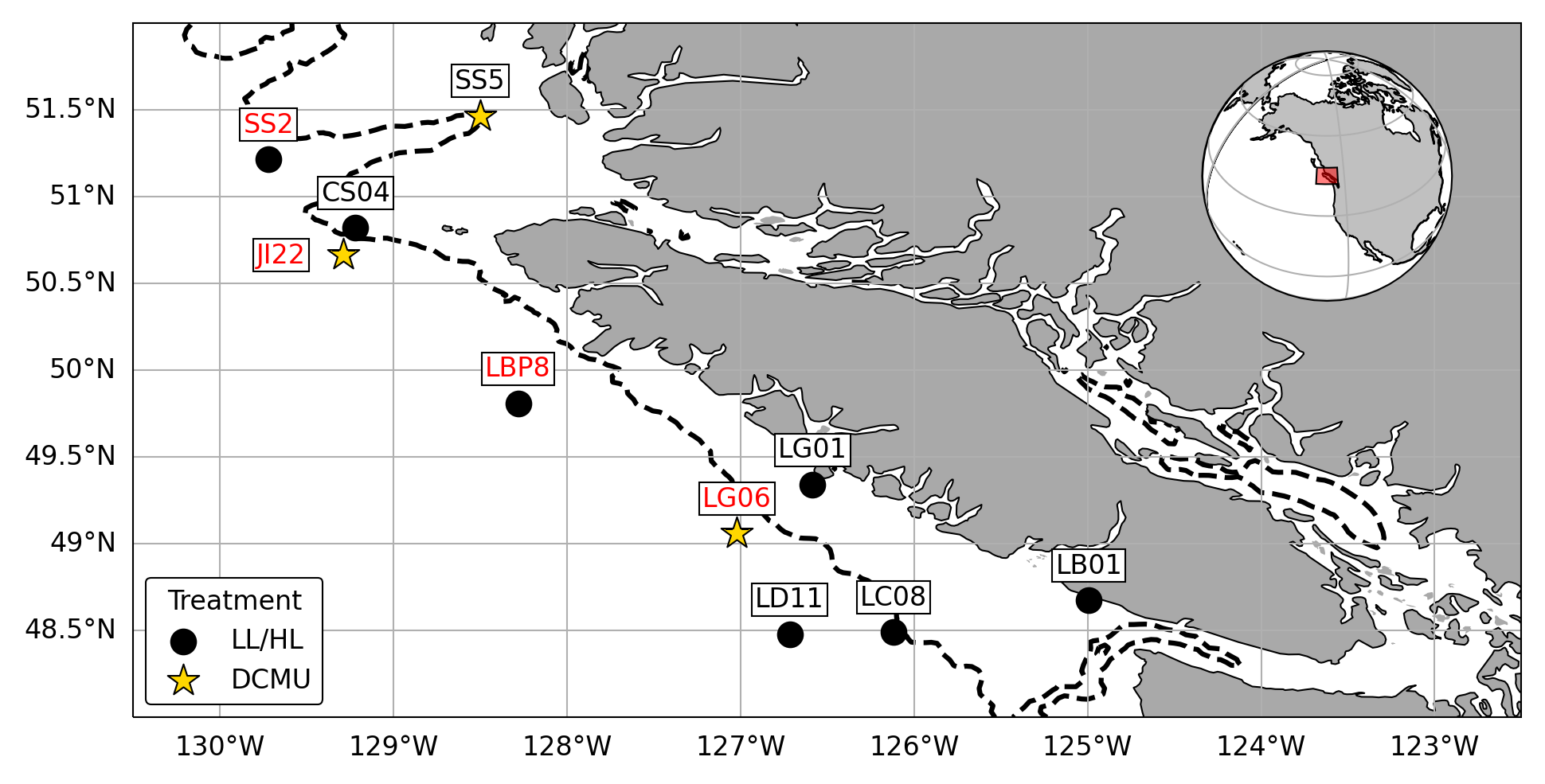

Supplement: S1 Fig — Markers indicate the type of experiments conducted; black circles denote light manipulations, while stars represent DCMU addition experiments. Red labels denote stations sampled in 2023. The dashed contour line indicates the 200 m depth contour, using bathymetry data obtained from US National Geophysical Data Center (NGDC) ETOPO2 dataset (retrieved from: https://rda.ucar.edu/datasets/d759003/). The inset map highlights the study region (red box) in the orthographic projection. These maps were produced using the “Cartopy” package (v0.21.1) in python (v3.8.16), with land features obtained from the Natural Earth dataset retrieved from the public domain (see https://www.naturalearthdata.com/about/). (TIF) [file pone.0317951.s001.tif]

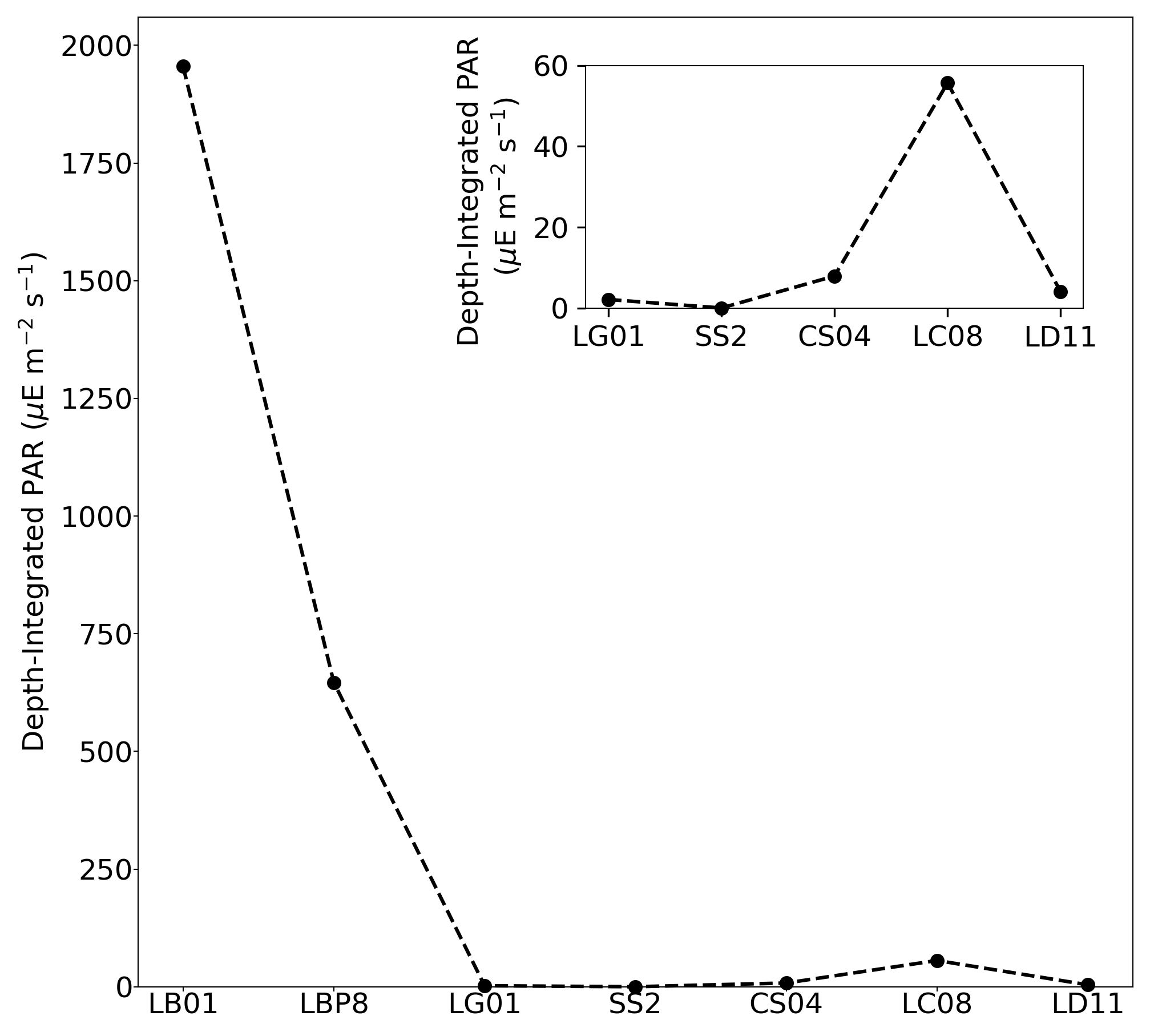

Supplement: S2 Fig — Note the order of stations is the same as Fig 2 in the main text. (TIF) [file pone.0317951.s002.tif]

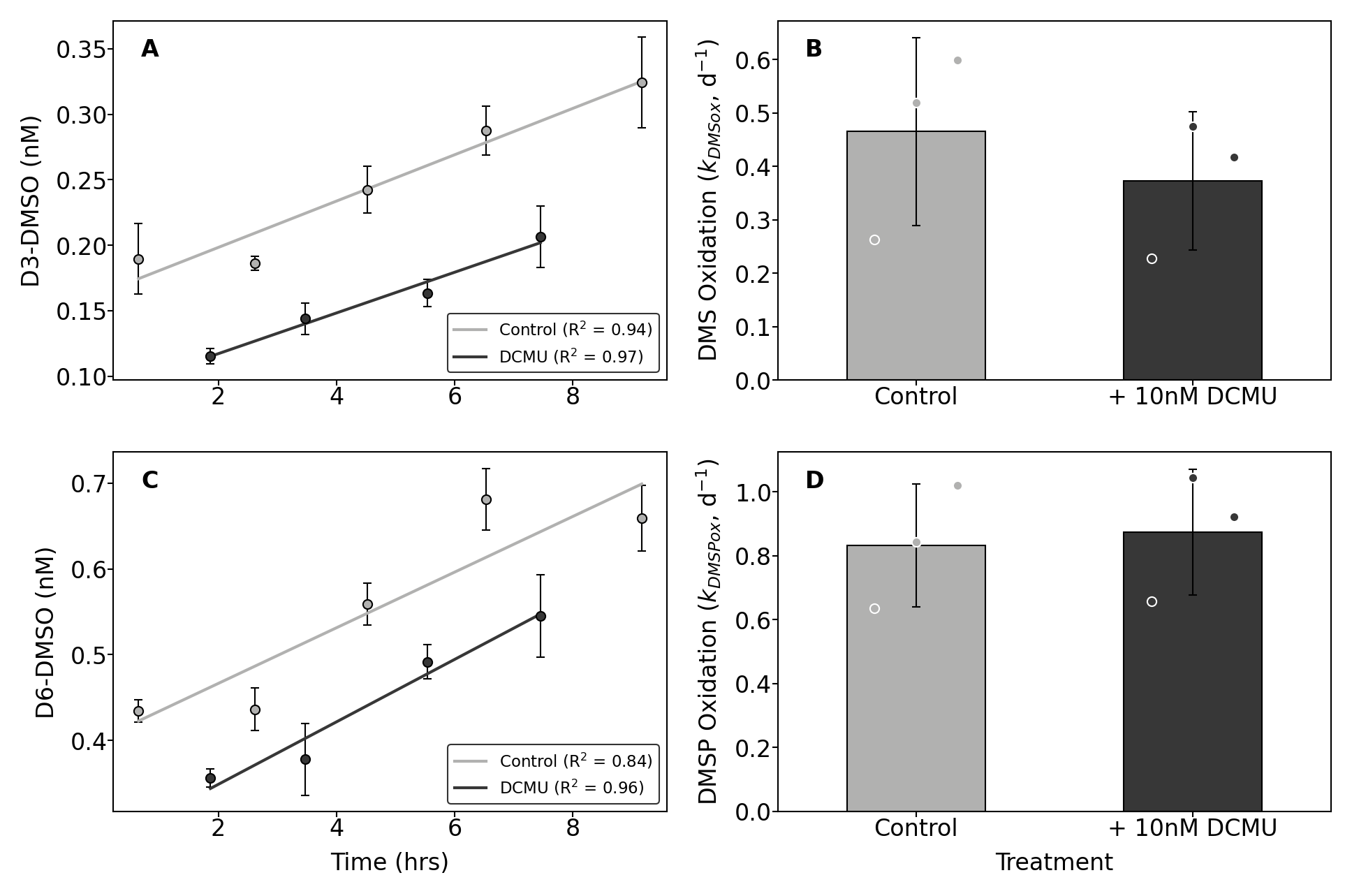

Supplement: S3 Fig — (A) Hourly yields of D3-DMSO (nM) derived from D3-DMS oxidation for control and 10 nM DCMU treatments. (B) Rate constants of D3-DMS oxidation (kDMSox, d-1) derived from the linear regression of time-course data shown in (A). Data points represent individual replicate measurements. (C) same as (A), for D6-DMSO formation from D6-DMSP oxidation. (D) same as (B) for rate constants of D6-DMSO formation (kDMSPox, d-1). All error bars indicate ± 1 s.d. (TIF) [file pone.0317951.s003.tif]

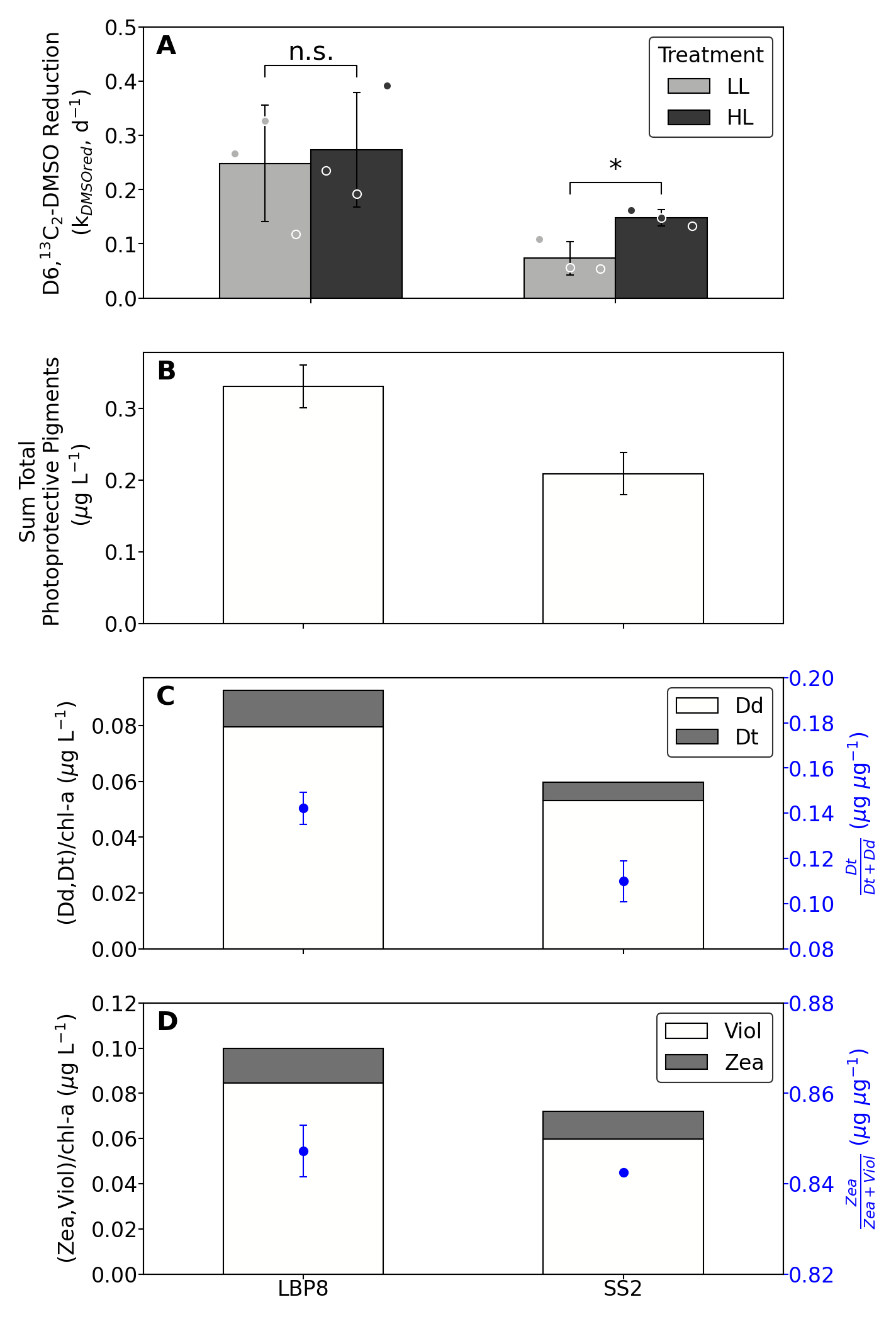

Supplement: S4 Fig — (A) Rate constants D6,13C2-DMSO reduction (kDMSOred., d-1; n = 3) for low light (LL) and high light (HL) treatments. Significance derived from two-tailed Student’s t-tests (*: p < 0.05, n.s.: non-significant), error bars denote ± 1 s.d. (B) Concentration of photoprotective pigments (μg L-1) obtained from HPLC analysis and calculated as the sum total of 9-cis-neoxanthin, violaxanthin, diadinoxanthin, diatoxanthin, alloxanthin, lutein, zeaxanthin, and β-carotene concentrations. (C) Concentrations of diadinoxanthin (Dd, μg L-1; light bars) and diatoxanthin (Dt, μg L-1; grey bars) normalized to total chlorophyll-a (chl-a, μg L-1) concentrations. Blue markers indicate the de-epoxidation ratios of the absolute concentrations of Dd and Dt. (D) Same as (C) for concentrations of zeaxanthin (Zea, μg L-1; light bars) and violaxanthin (Viol, μg L-1; grey bars) normalized to total chl-a, and their epoxidation ratios (blue markers). All error bars indicate range (n = 2) for pigment data. (TIF) [file pone.0317951.s004.tif]

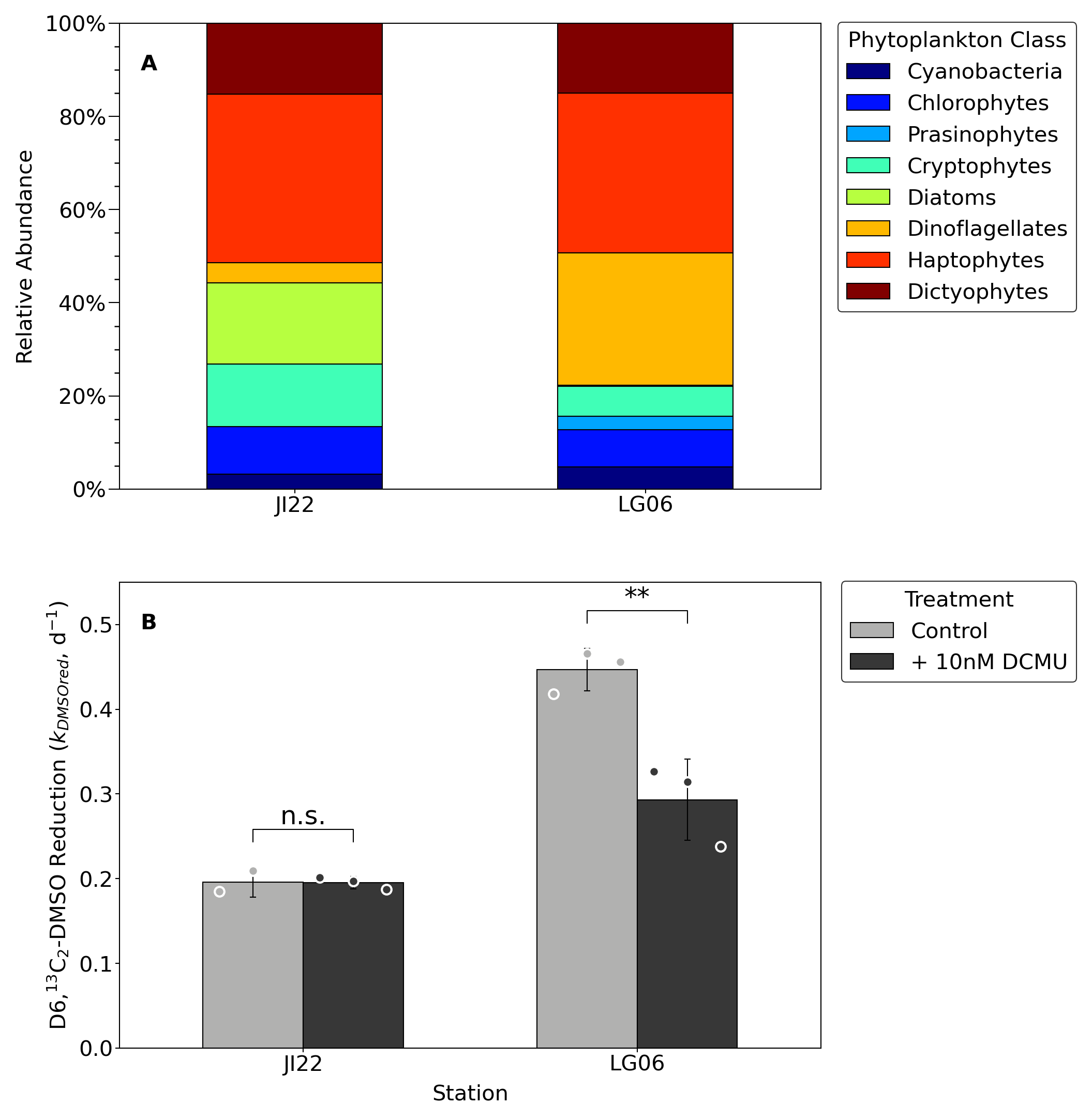

Supplement: S5 Fig — (A) Relative abundances (%) of different phytoplankton groups at stations JI22 and LG06 derived from CHEMTAX analysis. (B) Rate constants of D6,13C2-DMSO reduction (kDMSOred, d-1) in the control and +10 nM DCMU treatments for stations JI22 and LG06. Significance derived from two-tailed Student’s t-tests (**: p < 0.01, n.s.: non-significant). Error bars indicate range (n = 2) or ± 1 s.d. (n = 3). (TIF) [file pone.0317951.s005.tif]
