# Supplementary material for: A potential photo-protective, antioxidant function for DMSO in marine phytoplankton
Source: PLoS One. 2025 Feb 6;20(2):e0317951. doi: 10.1371/journal.pone.0317951 (PMC11801556; doi:10.1371/journal.pone.0317951)
Supplement: S1 Table — (DOCX) [file pone.0317951.s006.docx]

|  |  |  |  |  |  |
| --- | --- | --- | --- | --- | --- |
| Station | Treatment^†^ | Unlabelled DMS Turnover^§^ | Gross DMS  Consumption^§^ | DMSP Cleavage^§^ | DMSO Reduction^§^ |
| LB01^a^ | LL | 20.5 ± 4.9 (3)^‡^ | -0.66 ± 0.03 (3) | 0.82 ± 0.06 (3) | 0.45 ± 0.02 (3) |
|  | HL | 17.4 ± 3.5 (3) | -0.58 ± 0.14 (3) | 0.65 ± 0.23 (3) | 0.40 ± 0.04 (3) |
| LBP8^b^ | LL | -7.4 ± 3.7 (3) | -0.96 ± 0.2 (3) | 0.24 | 0.25 ± 0.11 (3) |
|  | HL | -4.3 ± 2.3 (3) | -0.99 ± 0.12 (3) | -0.36 | 0.27 ± 0.11 (3) |
| LG01^a^ | LL | 2.9 ± 0.29 (2) | b.d. | 0.65 ± 0.16 (3) | 0.15 ± 0.06 (3) |
|  | HL | 2.8 ± 0.57 (3) | b.d. | 0.71 ± 0.14 (3) | 0.13 ± 0.08 (3) |
| SS2^b^ | LL | -0.11 ± 1.4 (2) | b.d. | 0.27 ± 0.03* (3) | 0.07 ± 0.03* (3) |
|  | HL | -0.67 | -0.35 | 0.49 ± 0.05* (3) | 0.15 ± 0.01* (3) |
| CS04^a^ | LL | b.d. | -0.44 ± 0.23 (3) | 0.57 ± 0.02 (3)*** | 0.66 ± 0.08 (3)* |
|  | HL | 1.3 ± 0.42 (2) | -0.45 ± 0.04 (2) | 0.28 ± 0.04 (2)*** | 0.97 ± 0.11 (2)* |
| LC08^a^ | LL | 3.8 ± 0.29 (2) | -1.3 ± 0.3 (2) | 0.16 | 0.40 ± 0.02 (3)** |
|  | HL | 2.2 ± 2.8 (3) | -1.4 ± 0.25 (2) | 0.18 | 0.57 ± 0.05 (3)** |
| LD11^a^ | LL | 53.4 ± 31.3 (3) | -0.35 | 1.7 ± 0.4 (3) | 0.74 ± 0.95 (3)* |
|  | HL | 32.9 ± 3.7 (3) | -0.19 ± 0.14 (2) | 1.9 ± 0.4 (3) | 2.5 ± 0.2 (3)* |
| SS5^a^ | Control | b.d. | -0.32 ± 0.01 (2) | 0.38 ± 0.01 (3) | 0.99 ± 0.28 (3)* |
|  | DCMU | 0.01 ± 2.6 (2) | -0.34 ± 0.01 (2) | b.d. | 0.42 ± 0.04 (3)* |
| LG06^b^ | Control | -16.0 ± 4.7 (3)*** | -0.72 ± 0.02 (3) | 0.58 ± 0.07 (2) | 0.45 ± 0.02 (3)** |
|  | DCMU | -1.5 ± 1.8 (3)*** | -0.70 ± 0.05 (3) | 0.30 ± 0.10 (2) | 0.29 ± 0.05 (3)** |
| JI22^b^ | Control | 4.2 | -0.41 | 0.14 | 0.20 ± 0.02 (2) |
|  | DCMU | 5.1 ± 1.7 (3) | -0.17 | 0.21 | 0.20 ± 0.01 (3) |

^†^HL = high light (50% transmittance); LL = low light (1% transmittance); DCMU = 10 nM DCMU addition in HL; Control = HL only.

^§^Isotopic tracers measured by corresponding mass-to-charge ratios of 63 m/z (unlabelled DMS turnover), 66 m/z (gross D3-DMS consumption), 69 m/z (D6-DMSP cleavage), and 71 m/z (D6,^13^C_2_-DMSO reduction).

^a^Data collected in 2022.

^b^Data collected in 2023.

^‡^The number of replicates (n) are denoted in parentheses. ± value indicate range (n = 2) or 1 s.d. (n = 3).

b.d. = all replicate *k* values below the detection limit (see Methods).

Significance between treatments derived from two-tailed Student's t-tests (*: p < 0.05; **: p < 0.01; ***: p < 0.001).
