# Supplementary material for: A potential photo-protective, antioxidant function for DMSO in marine phytoplankton
Source: PLoS One. 2025 Feb 6;20(2):e0317951. doi: 10.1371/journal.pone.0317951 (PMC11801556; doi:10.1371/journal.pone.0317951)
Supplement: S2 Table — (DOCX) [file pone.0317951.s007.docx]

|  |  |  |  |  |  |  |  |  |  |  |  |  |  |  |  |  |
| --- | --- | --- | --- | --- | --- | --- | --- | --- | --- | --- | --- | --- | --- | --- | --- | --- |
| Station | Treatment^†^ | Unlabelled DMS Turnover Rate Constants^§^ | Gross DMS Consumption Rate Constants^§^ | DMSP Cleavage Rate Constants^§^ | DMSO Reduction Rate Constants^§^ | DMS Oxidation Rate Constants^‡^ | DMSP Oxidation Rate Constants^‡^ | Temperature (CTD) | Salinity (CTD) | Fluorescence (CTD) | Photosynthetically Active Radiation (PAR; CTD) | Mixed Layer Depth  (MLD; CTD) | Non-Photochemical Quenching (NPQ) | Maximum Photosynthetic Efficiency (F_v_/F_m_) | Xanthophylls (HPLC)^*^ | Community Composition (HPLC)^*^ |
| LB01^a^ | CRL | x | x | x | x |  |  | x | x | x | x | x |  |  |  |  |
|  | HL | x | x | x | x |  |  | x | x | x | x | x |  |  |  |  |
| LBP8^b^ | CRL | x | x | x | x |  |  | x | x | x | x | x | x | x | x | x |
|  | HL | x | x | x | x |  |  | x | x | x | x | x | x | x |  |  |
| LG01^a^ | CRL | x | x | x | x |  |  | x | x | x | x | x |  |  |  |  |
|  | HL | x | x | x | x |  |  | x | x | x | x | x |  |  |  |  |
| SS2^b^ | CRL | x | x | x | x |  |  | x | x | x | x | x | x | x | x | x |
|  | HL | x | x | x | x |  |  | x | x | x | x | x | x | x |  |  |
| CS04^a^ | CRL | x | x | x | x |  |  | x | x | x | x | x |  |  |  |  |
|  | HL | x | x | x | x |  |  | x | x | x | x | x |  |  |  |  |
| LC08^a^ | CRL | x | x | x | x |  |  | x | x | x | x | x |  |  |  |  |
|  | HL | x | x | x | x |  |  | x | x | x | x | x |  |  |  |  |
| LD11^a^ | CRL | x | x | x | x |  |  | x | x | x | x | x |  |  |  |  |
|  | HL | x | x | x | x |  |  | x | x | x | x | x |  |  |  |  |
| SS5^a^ | CRL | x | x | x | x | x | x | x | x | x | x | x |  |  |  |  |
|  | DCMU | x | x | x | x | x | x | x | x | x | x | x |  |  |  |  |
| LG06^b^ | CRL | x | x | x | x |  |  | x | x | x | x | x | x | x | x | x |
|  | DCMU | x | x | x | x |  |  | x | x | x | x | x | x | x |  |  |
| JI22^b^ | CRL | x | x | x | x |  |  | x | x | x | x | x | x | x | x | x |
|  | DCMU | x | x | x | x |  |  | x | x | x | x | x | x | x |  |  |
| ^†^HL = high light (50% transmittance); LL = low light (1% transmittance); DCMU = 10 nM DCMU addition in HL; Control = HL only. | | | | | | | | | | | | | | | | |
| ^§^Isotopic tracers measured by corresponding mass-to-charge ratios of 63 m/z (unlabelled DMS turnover), 66 m/z (gross D3-DMS consumption), 69 m/z (D6-DMSP cleavage), and 71 m/z (D6,^13^C_2_-DMSO reduction). | | | | | | | | | | | | | | | | |
| ^‡^Oxidation rates are measured as the formation of DMSO from D3-DMS (66 m/z, D3-DMSO) and D6-DMSP (69 m/z, D6-DMSO). | | | | | | | | | | | | | | | | |
| ^a^Data collected in 2022. | | | | | | | | | | | | | | | | |
| ^b^Data collected in 2023. | | | | | | | | | | | | | | | | |
| ^*^Samples collected prior to start of incubation treatments (HL or DCMU). | | | | | | | | | | | | | | | | |
