# Supplementary material for: A potential photo-protective, antioxidant function for DMSO in marine phytoplankton
Source: PLoS One. 2025 Feb 6;20(2):e0317951. doi: 10.1371/journal.pone.0317951 (PMC11801556; doi:10.1371/journal.pone.0317951)
Supplement: Attachment — Submitted filename: Review of manuscript PONE.docx [file pone.0317951.s008.docx]

**Review of manuscript PONE-D-24-45077**

**A photo-protective antioxidant function for DMSO in marine phytoplankton**

I have reviewed the above manuscript submitted by McNabb and Tortell regarding experiments conducted on a cruise that examined the response of DMSO reduction with respect to both light treatments and DCMU application. Their conclusions are that DMSO scavenges excess electrons coming from PSII in a secondary photoprotective response. I find that the results presented directly address the hypothesis and support it strongly. Their conclusions are not too speculative and they clearly articulate potential confounding factors. Their work is timely and provides new and interesting light on the DMS/O/P system in marine phytoplankton.

I do not have substantial criticism of their approach, as the methodology is sound, particularly for the experiments conducted. The triple isotope tracer method is ideally suited to teasing apart these various pathways.

The strongest criticism I can level at the paper is in reference to the NPR results present in Fig. 2. While the correlation (r = -0.53, n = 33) is statistically significant, it is very difficult to discern a true trend among the data presented. Partially this may be due to mixing two different treatment groups (DCMU and light), and it may also be due to the relatively large error bars on the points, particularly for NPQ measurements. There is likely a better way to present these data to show this relationship more clearly. It is also unclear to me why there are eight points per station. Of everything presented, this was probably the least clearly supportive of the overall conclusions.

**Specific suggestions**

Line 61: the authors include DMS as a potential antioxidant in cells, despite results showing that DMS is not present at sufficiently high concentrations to effectively scavenge ROS (ref. 56)

Line 76: there is a superscript 3 here. Not sure what that refers to

Lines 160-170: This might be the proper place to define both Dd and Dt, which appear later but should be described in this methods section

Line 243-244: Why just these two sites?

Line 251-253: The shading of the figure is a bit confusing. I initially looked at the figure and thought I was looking at low-light adapted (shaded) versus high-light adapted. This was not correct. I would recommend getting rid of the background shading so as to eliminate/reduce confusion.

Fig. 1F: I’m not certain that this panel actually contributes to the paper.

Lines 391-403: At this point, the authors begin to describe DMSO as part of a larger antioxidant system, linking it to DMSOP. Does this quantitatively hold water? How does the rate of DMSO reduction compare to DMSOP cleavage (even a rough estimate)? Does DMS exit the chloroplast or cell fast enough to not be subsequently oxidized? There is a lot that could be done here, and I leave it to the authors to decide how much to expand.
