# Supplementary material for: A potential photo-protective, antioxidant function for DMSO in marine phytoplankton
Source: PLoS One. 2025 Feb 6;20(2):e0317951. doi: 10.1371/journal.pone.0317951 (PMC11801556; doi:10.1371/journal.pone.0317951)
Supplement: Attachment — Submitted filename: PLOS Review.docx [file pone.0317951.s009.docx]

The study provides evidence that DMSO reduction by phytoplankton serves as a “secondary photo-protective” strategy that scavenges excess electrons produced between PSI and PSII in linear electron transport. The authors explain that the mechanism is secondary in nature because it would be induced when primary mechanisms (NPQ, protective pigment production) are already downregulated, which helps explain the inverse relationships they observe with DMSO reduction and NPQ and xanthophyls. Enzymatic reduction of DMSO would be metabolically efficient and DMSO would be a readily available sink for excess electrons because it is likely produced within chloroplasts and is also highly membrane permeable according to the literature. The authors describe the importance of this strategy in rapid vertical mixing events which helps explain prior evidence of correlations between DMS production and vertical mixing. Their field incubations also provide contextual evidence to support this physical phenomenon. I think the study is interesting and mostly well explained. I especially appreciate contextualizing the photoprotective strategy within the physical environment which boosts the interdisciplinary attributes of the study.

Major comments:

The data mostly support the conclusions except that there is inconsistency between DMSO reduction and inhibiting linear electron transport in their DCMU incubations, wherein they find that DCMU decreased DMSO reduction at 2 stations (SS5, LG06) but had no effect at another station (JI22) (Table S1). Further, the JI22 results are not shown together with all the results from the DCMU incubations in the main figures, but rather in the supplementary. The authors should show results from all 3 incubations in Figure 3. The authors explain that the discrepancy between these results could be due to respiration by heterotrophs or differences in phytoplankton community composition. Specifically, they state that “DMSO reduction rates appear to be lower in phytoplankton assemblages dominated by high DMSP-producing prymnesiophytes (such as haptophytes), and higher in assemblages with greater proportions of dinoflagellates” (lines 426-428). They point out specifically that JI22 was haptophyte dominated, but both JI22 and LG06 are haptophyte dominated (Figure S5). There are more dinoflagellates at LG06 than JI22 though. The study would benefit greatly from culture experiments assessing DMSO reduction and photophysiology under the treatments used in the field incubations from representatives of the taxonomic groups. However, I don’t think it is necessary for the work presented, but I hope it is done in the future. Are there any physical differences between these stations that could explain the discrepancy? Are there any physical data for these stations at all? Are there any data on the community composition at SS5? On this note, it would be helpful to have a supplementary summary table of the ancillary data for each station. Because of this finding (and that photophysiology data appear limited to 4 out of 10 stations), I think there should be more careful/qualifying language used in the title such as including “possible/potential” or “plausible”. The authors do a pretty good job of using careful language in the results and discussion, but I recommend combing through the manuscript again and using caution.

The methods describe that the “carboys were kept shielded during filling to minimize ambient light exposure and held for up to 6 hours in the dark at 4^o^C prior to the start of experiments” (lines 11-112). 6 hours of dark adaptation seems like a very long time for assessing photophysiology at least. Why was the water dark adapted for so long?

Lines 301 – 305 – Can the authors provide additional evidence or explanation of primary versus secondary photoprotective mechanisms? This section needs more explanation. Later text (lines 438-448, 456 – 460) describe the potential relevance of this in nature, however, it could help their explanations to briefly mention this here as well.

Figure 1a – please show both positive and negative error bars

Minor comments to take or leave:

Line 43-45 – suggest rewording for improved clarity

I suggest reminding the reader that pigments were sampled for prior to starting incubations before lines 279-282. At this point in the text, they may expect the opposite relationship if they do not read the methods. This may also help readers follow the authors’ later interpretations of the inverse relationship with DMSO reduction and protective pigments.
