# Supplementary material for: A potential photo-protective, antioxidant function for DMSO in marine phytoplankton
Source: PLoS One. 2025 Feb 6;20(2):e0317951. doi: 10.1371/journal.pone.0317951 (PMC11801556; doi:10.1371/journal.pone.0317951)
Supplement: Attachment — Submitted filename: Response to Reviewers.docx [file pone.0317951.s010.docx]

**Response to Reviewer #1:**

**Review of manuscript PONE-D-24-45077**

**A photo-protective antioxidant function for DMSO in marine phytoplankton**

I have reviewed the above manuscript submitted by McNabb and Tortell regarding experiments conducted on a cruise that examined the response of DMSO reduction with respect to both light treatments and DCMU application. Their conclusions are that DMSO scavenges excess electrons coming from PSII in a secondary photoprotective response. I find that the results presented directly address the hypothesis and support it strongly. Their conclusions are not too speculative and they clearly articulate potential confounding factors. Their work is timely and provides new and interesting light on the DMS/O/P system in marine phytoplankton.

I do not have substantial criticism of their approach, as the methodology is sound, particularly for the experiments conducted. The triple isotope tracer method is ideally suited to teasing apart these various pathways.

Thank you for your comments and feedback. We have addressed each specific concern below.

The strongest criticism I can level at the paper is in reference to the NPR results present in Fig. 2. While the correlation (r = -0.53, n = 33) is statistically significant, it is very difficult to discern a true trend among the data presented. Partially this may be due to mixing two different treatment groups (DCMU and light), and it may also be due to the relatively large error bars on the points, particularly for NPQ measurements. There is likely a better way to present these data to show this relationship more clearly. It is also unclear to me why there are eight points per station. Of everything presented, this was probably the least clearly supportive of the overall conclusions.

We have revised Fig. 2 to include separate panels showing the results partitioned by individual station and experimental treatment and an additional panel showing the pooled data with a single symbol type to illustrate the overall trend between DMSO reduction and NPQ.

**Specific suggestions**

Line 61: the authors include DMS as a potential antioxidant in cells, despite results showing that DMS is not present at sufficiently high concentrations to effectively scavenge ROS (ref. 56)

We have clarified this point to note that while DMS has antioxidant properties, its intracellular antioxidant capabilities are limited due to its likely rapid diffusion out of cells, as shown by Spiese et al. (2016). Spiese et al. (2016) argue antioxidant roles for DMS in cell membranes may still be significant for those phytoplankton species that are capable of significant DMSP synthesis and cleavage, and we have mentioned this caveat also in the revised manuscript in L65-68.

Line 76: there is a superscript 3 here. Not sure what that refers to

Thank you for catching this. This was a formatting typo that has now been corrected.

Lines 160-170: This might be the proper place to define both Dd and Dt, which appear later but should be described in this methods section

We have now defined Dd and Dt here and added some information about what they represent in L198-L204.

Line 243-244: Why just these two sites?

Unfortunately, community composition data are not available for the 2022 cruise. Instrumentation issues early during the 2023 cruise, combined with some sites being dedicated to DCMU experiments, restricted the spatial coverage of irradiance manipulation experiments to these two sites for comparison. At the request of another reviewer, we have also added a supplementary table (S2 Table) clarifying data availability for these cruises.

Line 251-253: The shading of the figure is a bit confusing. I initially looked at the figure and thought I was looking at low-light adapted (shaded) versus high-light adapted. This was not correct. I would recommend getting rid of the background shading so as to eliminate/reduce confusion.

We have now removed the shading to improve clarity.

Fig. 1F: I’m not certain that this panel actually contributes to the paper.

We find this panel helpful to illustrate conceptually how physical forcing may drive the biological responses described in text. We believe that some readers may also appreciate this figure and we are inclined to keep this panel. However, we are open to the editor’s recommendation on this point, noting that there are relatively few figures in the main text at present.

Lines 391-403: At this point, the authors begin to describe DMSO as part of a larger antioxidant system, linking it to DMSOP. Does this quantitatively hold water? How does the rate of DMSO reduction compare to DMSOP cleavage (even a rough estimate)? Does DMS exit the chloroplast or cell fast enough to not be subsequently oxidized? There is a lot that could be done here, and I leave it to the authors to decide how much to expand.

The reviewer’s comment raises some interesting and important points. In response, we have further expanded this section (L416-421) to include discussion on the diffusion rates of DMS, which are significant enough to limit its intracellular oxidative potential.

The contribution of DMSOP cleavage to DMSO formation is difficult to address quantitatively, as there is not enough data to provide a reasonable estimate of DMSOP cleavage rates. We have now acknowledged this in L425-430, and noted that evidence of DMSP storage in chloroplasts (and previous work calculating a high oxidative potential for DMSP with ROS) suggests that DMSOP formation and cleavage is a plausible source of intracellular DMSO in chloroplasts. The work of Thume et al. (2018) remains the only study yet to report on DMSO formation through DMSOP cleavage, but their experiments used DMSOP enrichments at several orders of magnitude above natural concentrations, and thus likely do not reflect *in situ* rates.

We also note that inferring the role of DMSOP from our measurements is not possible for the following reasons:

1) Our D6-DMSP oxidation rates reflect some combination of DMSP usage through either a) DMSOP formation / cleavage and b) cleavage to DMS and subsequent DMS oxidation to DMSO (Fig. 3D). The methods we used are unfortunately not equipped to tease apart the contributions of these separate pathways to net D6-DMSO formation with any confidence.
2) The D6-DMSP turnover also probably reflects mostly bacterial, rather than eukaryotic, metabolism, as intracellular uptake of dissolved DMSP is limited to certain species.

**Response to Reviewer #2:**

The study provides evidence that DMSO reduction by phytoplankton serves as a “secondary photo-protective” strategy that scavenges excess electrons produced between PSI and PSII in linear electron transport. The authors explain that the mechanism is secondary in nature because it would be induced when primary mechanisms (NPQ, protective pigment production) are already downregulated, which helps explain the inverse relationships they observe with DMSO reduction and NPQ and xanthophyls. Enzymatic reduction of DMSO would be metabolically efficient and DMSO would be a readily available sink for excess electrons because it is likely produced within chloroplasts and is also highly membrane permeable according to the literature. The authors describe the importance of this strategy in rapid vertical mixing events which helps explain prior evidence of correlations between DMS production and vertical mixing. Their field incubations also provide contextual evidence to support this physical phenomenon. I think the study is interesting and mostly well explained. I especially appreciate contextualizing the photoprotective strategy within the physical environment which boosts the interdisciplinary attributes of the study.

Thank you for your comments and feedback. We have addressed each specific concern below.

Major comments:

The data mostly support the conclusions except that there is inconsistency between DMSO reduction and inhibiting linear electron transport in their DCMU incubations, wherein they find that DCMU decreased DMSO reduction at 2 stations (SS5, LG06) but had no effect at another station (JI22) (Table S1). Further, the JI22 results are not shown together with all the results from the DCMU incubations in the main figures, but rather in the supplementary. The authors should show results from all 3 incubations in Figure 3.

We have now revised Fig. 3 to include the non-significant DCMU results from station JI22.

The authors explain that the discrepancy between these results could be due to respiration by heterotrophs or differences in phytoplankton community composition. Specifically, they state that “DMSO reduction rates appear to be lower in phytoplankton assemblages dominated by high DMSP-producing prymnesiophytes (such as haptophytes), and higher in assemblages with greater proportions of dinoflagellates” (lines 426-428). They point out specifically that JI22 was haptophyte dominated, but both JI22 and LG06 are haptophyte dominated (Figure S5). There are more dinoflagellates at LG06 than JI22 though. The study would benefit greatly from culture experiments assessing DMSO reduction and photophysiology under the treatments used in the field incubations from representatives of the taxonomic groups. However, I don’t think it is necessary for the work presented, but I hope it is done in the future.

We agree that culture studies would help elucidate the uncertainty as to the phytoplankton taxa(s) that utilize this physiological mechanism. Our group is considering future culture studies to expand on the findings of this study.

Are there any physical differences between these stations that could explain the discrepancy? Are there any physical data for these stations at all? Are there any data on the community composition at SS5?

Depths and ranges of the haloclines and thermoclines at LG06 and JI22 were comparable, as were the mixed layer depths (LG06: 7.9 m, JI22: 8.9 m). We have made a note of this on L458-459. We unfortunately do not have community composition data available for station SS5.

On this note, it would be helpful to have a supplementary summary table of the ancillary data for each station.

We have now added a supplementary S2 Table summarizing data availability for both cruises.

Because of this finding (and that photophysiology data appear limited to 4 out of 10 stations), I think there should be more careful/qualifying language used in the title such as including “possible/potential” or “plausible”. The authors do a pretty good job of using careful language in the results and discussion, but I recommend combing through the manuscript again and using caution.

We have updated the title to add “potential”, and further tempered the language in L439.

The methods describe that the “carboys were kept shielded during filling to minimize ambient light exposure and held for up to 6 hours in the dark at 4^o^C prior to the start of experiments” (lines 11-112). 6 hours of dark adaptation seems like a very long time for assessing photophysiology at least. Why was the water dark adapted for so long?

The long dark acclimation period was necessary due to logistical factors. Station arrival times (and water collection) were not fixed each day, but occurred at various times of day. Since both the light and DCMU experimental designs were conducted through daylight hours, we had to choose stations where bottle casts would be conducted early enough in the day to allow sufficient time to set up experiments (e.g. water collection, instrument calibration, prep of isotope working standards) and permit an 8-10 hour incubation window before sunset. In some cases, we had to choose between sampling 6 hours before dawn, or sampling from a station later in the morning. The latter would not have allowed for enough time to complete full experiments prior to sunset.

Lines 301 – 305 – Can the authors provide additional evidence or explanation of primary versus secondary photoprotective mechanisms? This section needs more explanation. Later text (lines 438-448, 456 – 460) describe the potential relevance of this in nature, however, it could help their explanations to briefly mention this here as well.

We have briefly expanded this section (L313 – 322) to include examples of both primary and secondary photoprotective mechanisms, and a more detailed explanation of why cells utilize secondary photoprotection mechanisms.

Figure 1a – please show both positive and negative error bars

We have updated all relevant figures to show negative error bars.

Minor comments to take or leave:

Line 43-45 – suggest rewording for improved clarity

This has now been reworded.

I suggest reminding the reader that pigments were sampled for prior to starting incubations before lines 279-282. At this point in the text, they may expect the opposite relationship if they do not read the methods. This may also help readers follow the authors’ later interpretations of the inverse relationship with DMSO reduction and protective pigments.

We have added a reminder that these samples were taken prior to the start of incubations on L294.
