# Supplementary material for: A potential photo-protective, antioxidant function for DMSO in marine phytoplankton
Source: PLoS One. 2025 Feb 6;20(2):e0317951. doi: 10.1371/journal.pone.0317951 (PMC11801556; doi:10.1371/journal.pone.0317951)
Supplement: Attachment — Submitted filename: Response to Reviewers.docx [file pone.0317951.s011.docx]

Reviewer #1:

I have read the above manuscript and find it much improved from the original draft. I do, however, still have some comments to make – one minor and one more involved.
The minor comment is an apparent typo on L199, “diadinoxanthin (Dt) and diadinoxanthin (Dd)”. Later, on L298, Dt is reported as “diatoxanthin”.

Thank you for catching this, we have now corrected it.
The more involved comment regards the paragraph on L413-437. Beginning on L416, the authors argue that DMSO has sufficient polarity to restrict diffusive loss across the membrane, citing two studies. Unfortunately, this statement is not supported by those studies or many others. DMSO rapidly crosses cell membranes. Lavoie et al. [ref. 64] have an extensive discussion about the factors that may impact their model, particularly concerning the multiple membranes in the chloroplast, if that is where DMSO is located. Indeed, even the authors indicate a high membrane permeability for DMSO on L432.

Thank you for pointing this out. We have now removed the incorrect statement regarding DMSO diffusivity on L416-417.
In this same paragraph, I would suggest to the authors to be clearer about DMSP compartmentalization. According to ref. 67, DMSP is localized to the chloroplast, as indicated on L428. Unlike DMSO, DMSP is membrane-impermeable owing to its zwitterionic nature. This would suggest that oxidation to DMSO(P) is actually highly likely, providing a DMSO source in situ without the need to argue for uptake from bulk seawater (L433), unless (as the authors state) the phytoplankton in question lack significant DMSP production.

We have noted in L426-427 that DMSP requires zwitterion-selective transport mechanisms, which will enable intracellular DMSP accumulation inside chloroplasts. We have revised second half of this paragraph (L429-436) to clarify that DMSP-DMSO redox is likely major source of intracellular DMSO, and clarified that uptake of dissolved DMSO is likely only necessary in non-DMSP synthesizing species.
